# Prognostic Impact of HER2 Low Status in Male Breast Cancer: Prospective Cohort Analysis

**DOI:** 10.3390/cancers16193399

**Published:** 2024-10-05

**Authors:** Atanas Ignatov, Sina Lempfer, József Mészáros, Holm Eggemann

**Affiliations:** 1 Department of Gynecology and Obstetrics, Otto-von-Guericke University, 39108 Magdeburg, Germany; jozsef.meszaros@med.ovgu.de; 2 Department of Gynecology and Obstetrics, Klinikum Magdeburg, 39108 Magdeburg, Germany; sina.lempfer@klinikum-magdeburg.de (S.L.);

**Keywords:** male breast cancer, HER2 low, HER2, survival

## Abstract

**Simple Summary:**

Male breast cancer (MBC) is a rare condition, and the role of low HER2 expression (HER2 low) is not well understood. In this prospective study, 870 MBC patients treated between May 2009 and June 2023 were evaluated to investigate the prognostic significance of HER2 low status. After a median follow-up of 43 months, 659 patients were classified into three groups: 76% were HER2 low, 12.3% were HER2 zero, and 11.7% were HER2 positive. HER2 positivity was linked to younger age, higher tumor proliferation, and more aggressive cancer characteristics, but no differences were found between HER2 zero and HER2 low groups. Furthermore, HER2 low status did not influence disease-free or overall survival. However, HER2 low has a potential clinical impact on MBC as a treatment target.

**Abstract:**

Background: Male breast cancer (MBC) is a rare disease, and the potential influence of low expression of human epidermal growth factor receptor 2 (HER2 low) remains unexplored. Methods: In this prospective cohort study, we evaluated 870 patients treated for MBC between May 2009 and June 2023 to assess HER2 low status and its prognostic implications. Results: With a median follow-up of 43 months (range 1–175 months), 659 eligible patients were categorized into three groups based on HER2 status: 501 (76%) HER2 low, 81 (12.3%) HER2 zero, and 77 (11.7%) HER2 positive. HER2 positivity correlated with younger age, higher proliferation index, non-specific type histology, lymphovascular invasion (LVSI), and low differentiation grade. Notably, all these parameters were equally distributed between the HER2 zero and HER2 low groups. Additionally, HER2 positivity was significantly associated with increased occurrences of regional and distant lymph nodes and pulmonary metastases. However, no statistically significant difference was observed between HER2 zero and HER2 low. Disease-free and overall survival showed no significant disparities between the groups. Conclusions: Our findings suggest that HER2 low status is frequently detected in MBC. Despite this, HER2 low did not correlate with clinical and pathological parameters, nor did it impact patients’ survival.

## 1. Introduction

Male breast cancer (MBC) is an uncommon disease, and its rarity makes the performance of prospective randomized trials very difficult [1]. Consequently, treatment approaches rely on limited retrospective studies and trials focused on managing female breast cancer (FBC). Emerging evidence suggests that MBC differs from FBC [1,2,3,4,5]. In a prospective cohort study, we demonstrated distinct clinical and pathological characteristics in MBC compared to FBC [1]. However, matching crucial characteristics between MBC and FBC indicated comparable survival rates for both [2].

The human epidermal growth factor receptor 2 (HER2) is a pivotal prognostic and predictive factor in both FBC and MBC. We have found that a moderate HER2 level is associated with distinct clinical behavior compared to HER2-negative tumors [6,7]. This finding is supported by numerous recent publications [8,9,10,11,12,13,14,15]. Notably, the HER2-directed antibody–drug conjugate T-DXd has exhibited efficacy in breast cancers, expressing lower levels of HER2 in prospective trials [16]. There is an increasing body of evidence that such therapy has significant potential in the treatment of HER2-expressing cancers [17,18,19,20,21]. This subset of tumors, characterized by lower HER2 expression (IHC scores of +1 or +2 with negative FISH test), has been termed “HER2-low” [22]. However, the prognostic relevance of HER2 low status in MBC remains unexplored.

Here, our objective is to examine the characteristics and survival outcomes of HER2 low MBC and compare them with HER2-negative and HER2-positive cases.

## 2. Patients and Methods

### 2.1. Patients

We conducted a study on male breast cancer (MBC) cases registered in Germany’s national prospective cancer registry, which is listed on the international clinical trial registry platform under the number DRKS00009536. This comprehensive database contains detailed information about MBC patients, including their diagnosis date, patient and tumor characteristics, surgical and neo-/adjuvant treatments, recurrence events, cause and date of death, secondary cancers, and comorbidities.

Our analysis included 870 men diagnosed with breast cancer between May 2009 and June 2023. We focused specifically on patients with non-metastatic invasive breast cancer. Cases were excluded if HER2 status was unspecified (*n* = 143), if the cancer was primary metastatic (*n* = 43), or if it was non-invasive (*n* = 25). HER2 status was assessed as previously described [23] using the Hercep Test™ (Agilent Technologies, D-76337 Waldbronn, Gemany) according to the manufacturer’s ins tructions. HER2 immunohistochemical (IHC) staining was evaluated based on intensity and distribution following ASCO/CAP guidelines. HER2 status was categorized as follows: HER2 zero, defined by an IHC score of 0; HER2 low, defined by an IHC score of 1+ or 2+ with negative in situ hybridization; and HER2 positive, defined by an IHC score of 3+ or 2+ with positive in situ hybridization.

This study adhered to ethical standards in accordance with the Declaration of Helsinki and Good Clinical Practice guidelines. Approval was obtained from the Research and Ethics Committee of Otto-von-Guericke University, Magdeburg, Germany. Informed consent was obtained from all study participants. The manuscript was prepared in line with the STROBE statement criteria [24].

### 2.2. Statistical Analysis

The statistical calculations were performed using SPSS version 29.0 (SPSS, Chicago, IL, USA). Clinical, pathological, and treatment parameters between groups were compared using the chi-squared test or the Fisher exact test for categorical variables and the two-sample *t*-test for age. Survival probability was estimated using the Kaplan–Meier method. The equality of survival curves was tested by the log-rank test. Disease-free survival (DFS) was defined as the period between the date of diagnosis to that of local and/or regional recurrence, distant metastases, or death from disease, whichever occurred first. Overall survival (OS) was defined as the time from the date of diagnosis to the date of death of any cause. The follow-up ends either with the patient’s death, the last available information, or the last follow-up on 1 June 2023. The statistical analyses were two sided, and *p*-values of less than 0.05 were considered statistically significant.

## 3. Results

**Figure 1:**
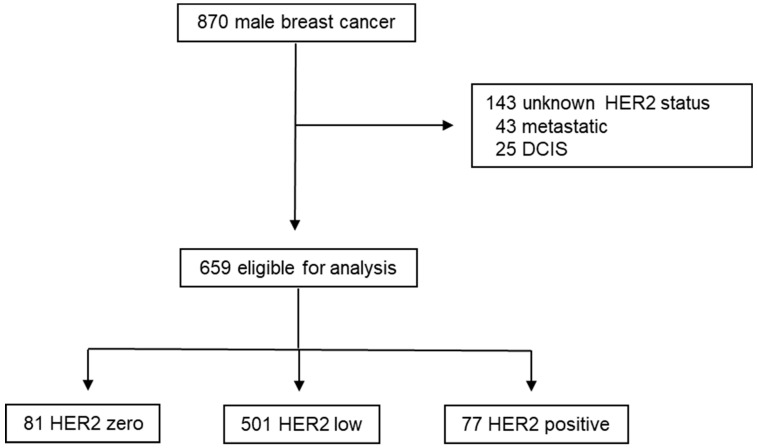
Study design.

The median follow-up was 43 months (range 1–175 months). Over the study period, 870 patients with breast cancer were included in the prospective register study. Of these, 213 were excluded from this analysis (Figure 1) due to metastatic disease (*n* = 43), non-invasive breast cancer (*n* = 23), and unknown HER2 status (*n* = 143).

Subsequently, 659 eligible male patients were categorized into three groups based on HER2 status: 501 (76%) were HER2 low, followed by 81 (12.3%) HER2 zero cases and 77 (11.7%) HER2 positive cases. Examination of various clinical parameters among these groups revealed significant differences (Table 1).

The median age of patients varies significantly across the three HER2 groups. Patients with zero HER2 expression have a median age of 68 years, with a range from 42 to 96 years. Those with low HER2 expression have a slightly higher median age of 70 years, ranging from 37 to 99 years. In contrast, patients with positive HER2 expression tend to be younger, with a median age of 65 years, and their ages range from 37 to 93 years. The difference in median age among the groups is statistically significant, with a *p*-value of 0.004.

Ki-67, a marker associated with cell proliferation, also shows a statistically significant variation across the HER2 groups. The median Ki-67 percentage is 15% (ranging from 3% to 60%) in the HER2 zero group, 20% (ranging from 4% to 80%) in the low HER2 group, and 28% (ranging from 8% to 73%) in the HER2-positive group. The *p*-value for this difference is 0.004, indicating a significant association between HER2 status and Ki-67 levels. The median BMI is relatively consistent across the groups, with HER2 zero at 27.7 (ranging from 22.1 to 45.4), low HER2 at 27.8 (ranging from 16.6 to 62.5), and HER2 positive also at 27.8 (ranging from 18.9 to 44.9). The *p*-value for BMI is 0.247, suggesting no significant difference in BMI across the different HER2 expression groups.

The distribution of histological subtypes shows significant variation between the groups. In the HER2 zero group, the majority of patients (87.7%) have no special type (NST) histology, with 1.2% having lobular histology and 11.1% classified as other types. For the low HER2 group, 93.6% have NST histology, 1.9% have lobular histology, and 4.6% fall into the other category. Notably, in the HER2-positive group, all patients (100%) have NST histology, with no cases of lobular or other histologies. The *p*-value for histological subtype distribution is 0.015, indicating a statistically significant difference across the HER2 groups.

Tumor status, categorized as T1 to T4, shows some variation across the groups, although it is not statistically significant (*p*-value of 0.543). In the HER2 zero group, 50.0% of patients are classified as T1, 35.0% as T2, 3.8% as T3, and 11.3% as T4. In the low HER2 group, 39.8% are T1, 46.6% are T2, 2.5% are T3, and 11.3% are T4. In the HER2-positive group, 37.3% are T1, 45.3% are T2, 2.7% are T3, and 14.7% are T4.

Nodal involvement is another critical parameter. In the HER2 zero group, 61.3% of patients are node negative, while 38.7% are node positive. In the low HER2 group, 55.9% are node negative and 44.1% are node positive. For the HER2-positive group, 48.7% are node negative and 51.3% are node positive. The *p*-value of 0.288 indicates that there is no statistically significant difference in nodal status between the HER2 groups.

LVSI shows a significant difference across the HER2 groups, with a *p*-value of 0.005. In the HER2 zero group, 64.9% of patients are LVSI negative, and 35.1% are LVSI positive. In the low HER2 group, 62.7% are LVSI negative, and 37.3% are LVSI positive. In contrast, in the HER2-positive group, only 42.6% are LVSI negative, while a higher proportion, 57.4%, are LVSI positive.

Tumor grading is highly variable and statistically significant across the HER2 groups, with a *p*-value of less than 0.001. In the HER2 zero group, 11.3% of tumors are grade 1, 77.5% are grade 2, and 11.3% are grade 3. In the low HER2 group, 8.0% are grade 1, 67.2% are grade 2, and 24.8% are grade 3. In the HER2-positive group, a lower percentage (5.1%) are grade 1, 50.0% are grade 2, and a notably higher percentage (44.9%) are grade 3.

Hormone receptor status does not differ significantly across the groups (*p*-value of 0.641). In the HER2 zero group, 97.5% of patients are HR positive and 2.5% are HR negative. The low HER2 group has 98.6% HR-positive and 1.4% HR-negative patients. Similarly, in the HER2-positive group, 97.5% are HR positive and 2.5% are HR negative. Once again, no difference was observed between HER2 zero and HER2 low.

Furthermore, we investigated the sites of recurrence between three groups. Local recurrence refers to the return of cancer at the original tumor site (Table 2).

Among patients with zero HER2 expression, there were no cases of local recurrence (0%). In the low HER2 group, 18 patients (3.6%) experienced local recurrence. The HER2-positive group had four patients (5.1%) with local recurrence. Although there is a difference in the percentages of local recurrence across the groups, the difference is not statistically significant, with a *p*-value of 0.162.

Furthermore, there were no regional recurrences (0%) in the HER2 zero group. There were no (0%) in the HER2 zero group. However, in the low HER2 group, eight patients (1.6%) had regional recurrences. The HER2-positive group had a higher rate, with four patients (5.1%) experiencing regional recurrence. This difference is statistically significant, with a *p*-value of 0.043, indicating that regional recurrence is more common in patients with positive HER2 expression.

Bone recurrence was observed in two patients (2.5%) in the HER2 zero group. In the low HER2 group, 20 patients (4.0%) had bone recurrences. The HER2-positive group had the highest percentage, with seven patients (8.9%) experiencing recurrence in the bones. Although there is an increasing trend in bone recurrence with higher HER2 expression, the difference is not statistically significant, with a *p*-value of 0.098.

In the HER2 zero group, two patients (2.5%) had pulmonary recurrences. The low HER2 group had nine patients (1.8%) with lung recurrences. The HER2-positive group showed a higher rate, with six patients (7.6%) experiencing pulmonary recurrence. This difference is statistically significant, with a *p*-value of 0.011, suggesting that HER2-positive patients are more likely to have pulmonary recurrences.

Hepatic recurrence was not observed in the HER2 zero group (0%). In the low HER2 group, seven patients (1.4%) experienced hepatic recurrence. The HER2-positive group had 2 patients (2.5%) with liver recurrences. However, the difference in hepatic recurrence across the HER2 groups is not statistically significant, with a *p*-value of 0.382.

Brain recurrence was not observed in the HER2 zero group (0%). In the low HER2 group, three patients (0.6%) had brain recurrences. In the HER2-positive group, two patients (2.5%) experienced recurrence in the brain. The difference in brain recurrence rates is not statistically significant, with a *p*-value of 0.130.

Lymphatic recurrence, involving the distant lymph nodes, was observed in two patients (2.5%) in the HER2 zero group. In the low HER2 group, three patients (0.6%) experienced lymphatic recurrence. The HER2-positive group had a significantly higher percentage, with four patients (5.1%) experiencing lymphatic recurrence. This difference is statistically significant, with a *p*-value of 0.004, indicating that HER2-positive patients are more likely to have lymphatic recurrences.

Recurrences in other unspecified sites were observed in one patient (1.3%) in the HER2 zero group, five patients (1.0%) in the low HER2 group, and one patient (1.3%) in the HER2-positive group. There is no significant difference in the rate of recurrence in other sites across the HER2 groups, as indicated by a *p*-value of 0.965.

This study further explored the correlation between HER2 status and various treatment strategies (Table 3).

HER2 status exhibited correlations with axillary surgery and systemic treatment. Axillary lymph node dissection (ALND) was more frequently employed in the HER2-positive group (33.3%) compared to the HER2 zero (17.3%) and HER2 low (28.6%) groups. Systemic treatment was predominantly administered in HER2-positive tumors (81.6%). The utilization of chemotherapy in the HER2 zero and HER2 low groups was 24.1% and 36.3%, respectively, revealing a statistically significant difference (*p* = 0.031). The surgery of the breast and the radiation of the breast and the axilla were equally distributed between the three groups (Table 3).

**Figure 2:**
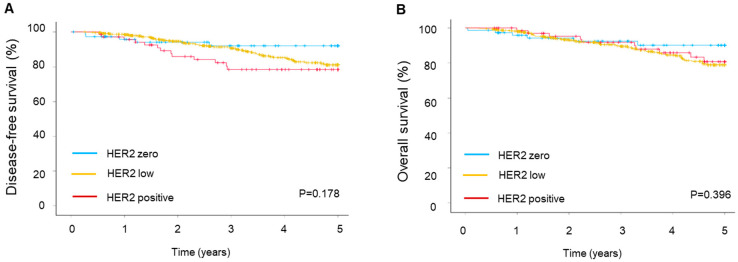
Survival outcomes depend on HER2 status. (**A**) Disease-free and (**B**) overall survival of male breast cancer patients.

Survival outcomes showed no significant differences between the groups. The 5-year DFS rates were 93.2% for patients in the HER2 zero group, 88.0% for those in the HER2 low group, and 81.2% in the HER2-positive group (Figure 2A). This was not statistically different between the groups (*p* = 0.178). Similarly, the 5-year OS rates were comparable between the two groups (Figure 2B, *p* = 0.396). The estimated 5-year OS rates were 91.9%, 85.8%, and 85.5% for the HER2 zero, HER2 low, and HER2-positive groups, respectively.

## 4. Discussion

This prospective study offers valuable insights into the correlation between HER2 low status and MBC. Within our prospectively recruited cohort, 76% of the tumors exhibited HER2 low status, 12.3% were HER2 zero, and 11.7% were HER2 positive. This positivity aligns closely with previous reports [1,25]. Unfortunately, there is a lack of reports on HER2 low status in MBC. Notably, the rate of HER2 low in FBC is significantly lower than what we observed in MBC [22]. This observation could be attributed to the higher frequency of HER2 low status in hormone receptor (HR)-positive FBC, considering that most MBCs are HR positive. In our cohort, over 97% of the tumors were HR positive. The prevalence of HR-positivity in our cohort likely accounts for the absence of a significant correlation between HER2 low status and HR status. Additionally, it seems that low HER2 expression is more frequently linked with markers indicating a favorable prognosis in FBC. On the contrary, Jacot et al. [26] demonstrated a significant correlation between HER2 low and low-grade tumors in triple-negative FBC. Our cohort did not show a significant correlation between HER2 low and tumor grading compared to HER2 zero status. Remarkably, HER2 low status did not exhibit a significant difference compared to HER2 zero status in our study.

Numerous studies have explored the association between HER2 low expression and patient outcomes in FBC. In a large cohort study using National Cancer Database data in the US, Peifer et al. found that HER2 low patients had a survival rate similar to those with HER2 zero tumors [27]. This was supported by other studies [26,28]. However, some reports suggest a favorable outcome for HER2 low compared to HER2 zero. Xu et al. reported similar disease-free survival (DFS) in ER-positive tumors for HER2 low and HER2 zero in the first 5 years after diagnosis, with better survival thereafter [29]. Better DFS and overall survival (OS) were also observed in a study involving 23,000 Asian patients [30]. Furthermore, a pooled analysis of neoadjuvant trials reported better DFS and OS with HER2 low status in HR-negative but not HR-positive patients [31].

The promising outcomes observed with anti-drug conjugates in breast cancer patients with low HER2 expression have stimulated extensive research efforts to understand the clinical characteristics of these tumors [22]. Ongoing studies are testing additional potent agents targeting HER2 as potential treatments for HER2 low breast cancer. One interesting treatment strategy is the use of antibody–drug conjugates (ADCs). The conjugate trastuzumab deruxtecan has been investigated in female breast cancer patients. In a prospective randomized trial, trastuzumab deruxtecan was associated with improved median DFS and OSl in patients with HER2 low metastatic breast cancer who had received one or two prior lines of chemotherapy [16] (Modi et al.). The effectiveness of this ADC was further confirmed in the DESTINY-Breast06 trial [32] (Bardia et al.). In this prospective randomized trial, trastuzumab deruxtecan resulted in longer progression-free survival compared to chemotherapy in patients with hormone receptor-positive, HER2 low, or HER2 ultralow metastatic breast cancer. These trials, along with our findings, impressively demonstrate the therapeutic potential of this drug in HER2 low MBC. Understanding the biology of HER2 low tumors in breast cancer is crucial for tailoring effective treatment strategies and enhancing our comprehension of the underlying biology of HER2-associated cancers. However, it is essential to interpret these findings in the context of the study limitations and the broader clinical landscape.

The primary limitation of our study is the small number of HR-negative patients, preventing a comparison of the effect of HR status as described for FBC. Additionally, HER2 status was not confirmed through a central pathological review. Nevertheless, this study boasts several strengths, including its prospective nature, well-maintained documentation, and population-based design with minimal exclusion criteria, resulting in a high level of external validity.

## 5. Conclusions

MBC exhibits a higher prevalence of HER2 low expression. HER2 low status in MBC does not show significant associations with clinical and pathological factors or patient outcomes. However, HER2 low remains an attractive option for HER2-targeted therapy.

## Figures and Tables

**Table 1:** Clinical and pathological parameters.

	HER2			
	Zero	Low	Positive	*p*-Value
Age, median	68 (42–96)	70 (37–99)	65 (37–93)	0.004
Ki-67%, median	15 (3–60)	20 (4–80)	28 (8–73)	0.004
BMI, median	27.7 (22.1–45.4)	27.8 (16.6–62.5)	27.8 (18.9–44.9)	0.247
Histo NST Lobular Other	71 (87.7%) 1 (1.2%) 9 (11.1%)	450 (93.6%) 9 (1.9%) 22 (4.6%)	74 (100%) 0 (0%) 0 (0%)	0.015
T status 1 2 3 4	40 (50.0%) 28 (35.0%) 3 (3.8%) 9 (11.3%)	193 (39.8%) 225 (46.6%) 12 (2.5%) 55 (11.3%)	28 (37.3%) 34 (45.3%) 2 (2.7%) 11 (14.7%)	0.543
N status Negative Positive	46 (61.3%) 29 (38.7%)	269 (55.9%) 212 (44.1%)	37 (48.7%) 39 (51.3%)	0.288
LVSI Negative Positive	48 (64.9%) 26 (35.1%)	288 (62.7%) 171 (37.3%)	29 (42.6%) 39 (57.4%)	0.005
Grading 1 2 3	98 (11.3%) 62 (77.5%) 9 (11.3%)	39 (8.0%) 328 (67.2%) 121 (24.8%)	4 (5.1%) 39 (50.0%) 35 (44.9%)	<0.001
HR status Negative Positive	2 (2.5%) 79 (97.5%)	7 (1.4%) 492 (98.6%)	2 (2.5%) 77 (97.5%)	0.641

**Table 2:** Recurrence site.

Recurrence Site	HER2			*p*-Value
	Zero	Low	Positive	
Local	0 (0%)	18 (3.6%)	4 (5.1%)	0.162
Regional	0 (0%)	8 (1.6%)	4 (5.1%)	0.043
OSS	2 (2.5%)	20 (4.0%)	7 (8.9%)	0.098
PUL	2 (2.5%)	9 (1.8%)	6 (7.6%)	0.011
HEP	0 (0%)	7 (1.4%)	2 (2.5%)	0.382
BRA	0 (0%)	3 (0.6%)	2 (2.5%)	0.130
LYM	2 (2.5%)	3 (0.6%)	4 (5.1%)	0.004
OTH	1 (1.3%)	5 (1.0%)	1 (1.3%)	0.965

**Table 3:** Treatment characteristics.

	HER2			
	Zero	Low	Positive	*p*-Value
Surgery BCS Mastectomy	73 (94.8%) 4 (5.2%)	463 (97.3%) 13 (2.7%)	72 (96.0%) 3 (4.0%)	0.475
Surgery of axilla SnB SnB + ALND ALND	54 (72.0%) 8 (10.7%) 13 (17.3%)	268 (59.0%) 56 (12.3%) 130 (28.6%)	30 (43.5%) 16 (23.2%) 23 (33.3%)	0.006
Radiation No Yes	13 (30.2%) 30 (69.8%)	75 (28.7%) 186 (71.3%)	5 (12.8%) 34 (87.2%)	0.101
Regional node irradiation No Yes	20 (50.0%) 20 (50.0%)	87 (43.7%) 112 (56.3%)	12 (31.6%) 26 (68.4%)	0.239
Systemic therapy No Yes	60 (75.9%) 19 (24.1%)	304 (63.7%) 173 (36.3%)	14 (18.4%) 62 (81.6%)	<0.001

## Data Availability

Data is unavailable due to ethical restrictions.
